# Editorial: Association Between Individuals’ Genomic Ancestry and Variation in Disease Susceptibility

**DOI:** 10.3389/fgene.2022.831320

**Published:** 2022-02-02

**Authors:** Ranajit Das, Tatiana V. Tatarinova, Elvira R. Galieva, Yuriy L. Orlov

**Affiliations:** ^1^ Yenepoya Research Centre, Yenepoya University, Mangalore, India; ^2^ Natural Science Division, La Verne University, La Verne, CA, United States; ^3^ Department of Fundamental Biology and Biotechnology, Siberian Federal University, Krasnoyarsk, Russia; ^4^ Life Sciences Department, Novosibirsk State University, Novosibirsk, Russia; ^5^ Institute of Cytology and Genetics SB RAS, Novosibirsk, Russia; ^6^ Agrarian and Technological Institute, Peoples’ Friendship University of Russia (RUDN University), Moscow, Russia; ^7^ Institute of Digital Medicine, I.M.Sechenov First Moscow State Medical University (Sechenov University), Moscow, Russia

**Keywords:** genetic ancestry and disease susceptibility, ancestry and genetic tapestry, ancestry-specific medicine, genomic profiling, genomic healthcare, personalized therapeutics, genome wide association study (GWAS)

This *Association between Individuals’ Genomic Ancestry and Variation in Disease Susceptibility* issue presents the studies related to the fields of human genetics, population genetics, and genetic and ancestral affiliations of various diseases. Our DNA determines not only who we are but also holds the key to uncovering our true ancestral past. A plethora of information stored inside the genome reflects our uniqueness and proximity to different ancestral and modern-day populations. Given the high correspondence between our genetic make-up and the geographical origin of our forefathers, it is possible to glean into precise ancestral origin using the genetic information. Understanding one’s ancestry is not only a ‘homing’ tool bringing someone closer to human evolutionary past but also holds the key in determining population-specific variability in disease susceptibility, drug responsiveness, and other health and fitness related traits.

Accordingly, we organized this Research Topic to collect the papers focused on studying ancestry specific variation in various human diseases. This Topic complements recent Research Topic *Bioinformatics of Genome Regulation* in *Frontiers in Genetics* considered more genetic background rather than molecular mechanisms of the human diseases (Orlov et al., 2021a).

Understanding one’s ancestry can play a monumental role in understanding variation in disease susceptibility across various populations and glean into the complex gene and environment interplay in ancestry-specific disorders. For instance, cardiovascular diseases tend to manifest in distinct ways unique to the ancestry of the patient such that people with high African ancestry proportion tend to have strokes as a result of cardiovascular disease, while people of South Asian ancestry tend to have heart attacks (Harshfield et al., 2021). Traditional high fat and protein diets in cold regions of Siberia and North Asia have consequences on obesity and diabetes-related diseases (Bai et al., 2015; Tiis et al., 2020). Individuals with larger fractions of Western Hunter Gatherer (WHG) related ancestry has been shown to develop more severe COVID-19 symptoms (Upadhyai et al., 2021).

In the realms of human population genetics, we strived to address similar questions in a global context, in an attempt to expand our existing knowledge to better understand the association between individuals’ genetic ancestry and disease susceptibility. This understanding can facilitate the development of novel therapeutics or repurposing of existing treatment strategies, particularly aiding in identifying population-specific therapies. Subsequently, our knowledge of ancestry-specific variation in complex disorders can be used towards developing personalized and ancestry-specific precision medicine approaches to ameliorate several complex disorders.

We considered the following themes for this special issue:

• Unravel predisposition of various modern-day populations towards common disorders and conditions, including but not limited to cancers, heart diseases, and infectious diseases.
• The association of alleles with complex disorders, evaluated at a population level; discovery of novel disease marker panels.
• Identification of novel medically relevant genetic variants that can be used as diagnostic markers in genetic diagnostics and healthcare.
• Selection dynamics of various genes. Investigation of the spatial and temporal distributions of positively selected alleles in response to population specific disease susceptibility.

The papers published in this Research Topic correspond to the themes stated above and extend the studies presented in *Frontiers in Genetics* Topics (see https://www.frontiersin.org/research-topics/17947/bioinformatics-of-genome-regulation-volume-ii). Recently we had organized series of conferences on human population genetics and computational genomics in Russia that allowed formalize the idea of special journal issues. The conference “Century of Human Population Genetics” was held in Moscow in 2019 (Tatarinova et al., 2020a). The conference was focused on the discussion of the research on gene pools of the world’s nations, ancient DNA analysis, possibilities of judicial genetics, population-genetic database development, biobanks, and new genomics technologies (Tatarinova et al., 2020b). We had series of publications on human ancestry based on new genomics data initially discussed at this meeting (Das et al., 2020; Orlov et al., 2021b). The BGRS\SB (Bioinformatics of Genome Regulation and Structure\Systems Biology) multi-conference (https://bgrssb.icgbio.ru/2020) has been organized in Novosibirsk, Russia in 2020 and was associated with the Research Topic on gene expression regulation in *Frontiers in Genetics* (see also https://www.frontiersin.org/research-topics/8383/bioinformatics-of-genome-regulation-and-systems-biology) (Orlov et al., 2016a, Orlov et al., 2016b, Tatarinova et al., 2019; Orlov et al., 2021a).

In this Research Topic a total of 11 papers could be arranged by two main areas - the human population genetics and ancestry studies, and the works on molecular mechanisms of the diseases.

Among the ancestry-based studies, Dashti et al. studied association of mtDNA haplogroups with obesity using high throughput sequencing technologies. Previous studies indicated that certain mtDNA variants and haplogroup lineages were associated with obesity. Dashti et al. presents the first study that used whole-exome data to extract entire mitochondrial haplogroups and consecutively study its association with obesity in an Arab population.

Susana Hernández-Doño et al. studied genetic determinants of systemic lupus erythematosus in Mexican population based on Human Leukocyte Antigen (HLA) haplotypes. Consistent with the admixture estimations, the origin of all risk alleles and haplotypes found in this study were found to be European, while the protection alleles were found to be Mexican Native American. Petrova et al. studied human genome variation in CFTR gene related to cystic fibrosis in the European and North Caucasian Part of Russia. The widespread introduction of technologies of whole genome and whole-exome analysis into practical healthcare has significantly increased the number of genetically determined diseases in the structure of human morbidity. Zinchenko et al. (2021) discussed the point (PP) and cumulative prevalence (PP), as well as the burden of the most common rare hereditary diseases (RHDs) (autosomal dominant, autosomal recessive, and X-linked) among 14 remote populations of the European part of Russia. Ramensky et al. studied genetic predisposition to cardiovascular diseases. The authors performed a targeted sequencing of 242 clinically important genes mostly associated with cardiovascular diseases.

The second set of papers contains the research articles, database application and the reviews highlighting genetic background and molecular mechanisms of the diseases. Similar studies have been discussed in previous issues on the topic (Snezhkina et al., 2020).

Kamenova et al. studied interactions of miRNA with mRNA in human genes associated with neurodegenerative diseases. Parkinson’s disease has complex genetic background that challenges bioinformatics methods for analysis of genes’ network and search for target genes (Orlov et al., 2021c). Recent studies have established a correlation between the disease and miRNA expression (Mukushkina et al., 2020). The authors described quantitative characteristics of the interactions between miRNAs and the mRNAs of candidate Parkinson’s disease genes. Myrzabekova et al. have studied potential effects of exogenous miRNA to human gene expression. The authors described potential human gene targets for such miRNA.

Tarasova et al. presented bioinformatics database on Human immunodeficiency virus (HIV) to study associations of clinical data of infected patients and viral sequences. Resistance of HIV to current drugs raises problems of treatment of this complex disease. The authors developed the RHIVDB database with free access that could help in drug target search.

Swart et al. studied genetic factors for developing active form of pulmonary *tuberculosis*, caused by *Mycobacterium tuberculosis*. The authors found *tuberculosis* susceptibility loci using local ancestry adjusted allelic association analysis. Naik et al. have reviewed genetic susceptibility to fungal infections in humans. Here, the authors discussed the polymorphisms in the genes of the immune system, the way it contributes toward some common fungal infections.

Gozman et al. highlighted the putative role of patients’ genetic background in response to the exposure to coronavirus. Hosts’ resistance to COVID-19 is thought to be related to the immune system. However, the genes are yet to be revealed. The variation in interferon genes to disease severity was discussed in this article. Gozman et al. raised important actual problem of the role of genetic variance in disease severity in COVID-19. Further, Prof Balanovsky et al. (2021) recently studied the variation of individual genomes associated with the severe COVID-19. This is a burning topic and the problem is continuing to be actively discussed (Upadhyai et al., 2021; Gerasimov et al., 2021; Lu et al., 2021) in relation to new data.

Overall, we are proud of the Research Topic at *Frontiers in Genetics* we collated. We hope that you will find this paper collection a stimulating reading and consider coming to the next conferences in this area in life format next year (https://bgrssb.icgbio.ru/2022/). The complementary Research Topics in *Frontiers* (https://www.frontiersin.org/research-topics/21036/high-throughput-sequencing-based-investigation-of-chronic-disease-markers-and-mechanisms) continues collection of papers on human diseases’ markers.
